# Osteosynthesis using plates and screws after removing a limited area of the periosteum in order to reduce misclassified during radiological assessment metacarpal shaft fractures

**Published:** 2016

**Authors:** TP Neagu, SA Popescu, C Cobilinschi, R Tincu, M Tiglis, I Lascar

**Affiliations:** * Department of Plastic Surgery and Reconstructive Microsurgery, Clinical Emergency Hospital of Bucharest, Romania; ** 11th Clinical Department, “Carol Davila” University of Medicine and Pharmacy Bucharest, Romania; *** Department of Anesthesiology and Intensive Care, Clinical Emergency Hospital of Bucharest, Romania; **** 14th Clinical Department, “Carol Davila” University of Medicine and Pharmacy Bucharest, Romania

**Keywords:** metacarpal, plates and screws, misclassified, DASH score

## Abstract

Hand fractures are one of the most common causes for presenting to the emergency room. Metacarpal fractures count about 18 to 44% of all hand fractures, and are most often standalone closed injuries, without misplacement, not needing operative treatment. We present a case in which osteosynthesis with plates and screws was used to reduce two metacarpal fractures in order to allow an early motion recovery, despite the fact that a small portion of the periosteum needed to be removed. The type of fractures were misclassified according to the radiological findings, therefore the correct diagnosis was established during surgery. The results according to the radiological aspects and to the DASH score were excellent with 95% function recovery at twelve months. In this case, the use of osteosynthesis with plates and screws led to a good fracture healing without any major complications. However, there are a series of complications related to this method that should be taken into consideration. Being misled by the radiological aspects of the fractures, the most certain way to classify a metacarpal shaft fracture is through exploratory surgery, even if in most of the cases the three radiological views are enough to establish the diagnosis.

**Abbreviations:** DASH score = Disability of Arm, Shoulder and Hand score, TAM = Total Active Motion, MCP = metacarpal phalangeal joint, PIP = proximal inter phalangeal joint

## Introduction

The hand is one of the most complex anatomical structures of the human body. It gives people the ability to feel and interact with their surroundings. The high density of nerves endings and various ways of positioning, offers unlimited tactile feedback of the things that are touched or grasped. Along with the seeing, hearing and smelling, the sense of touch gathers lots of information in order to create a brain projection of our environment. The actions performed by using hands are essential for life and survival. Therefore, every hand trauma it is perceived as being dramatic for the patient. During direct contact, or in order to avoid injuries to other parts of the body, hand fractures are one of the most common causes for presenting to the emergency room and within plastic surgery clinics. Metacarpal fractures counts about 18 to 44% of all small bone fractures [**1**,**2**]. These fractures are most often standalone injuries, without emerging through the skin and misplacement of the bone segments. At present, a guideline for the surgeon on the best available solution to treat this pathology is not well defined, even if there are many articles and studies regarding different outcomes of treatment protocols applied [**3**].

The treating physician can choose between the non-operative management of stable fractures and many osteosynthesis techniques for the treatment of the unstable or less stable fractures. Some of these surgical techniques involve the removal of a small portion of the periosteum from the bone in order to reduce the fracture and install the plates and screws, despite the fact that the periosteum plays an important role in the fracture healing. In this case report, reducing the fractures by using plates and screws and achieving sufficient stability to permit an early motion rehabilitation contra-balanced the effects on the fracture healing of the removal of a small portion of the periosteum.

## Case report

**Fig. 1A:**
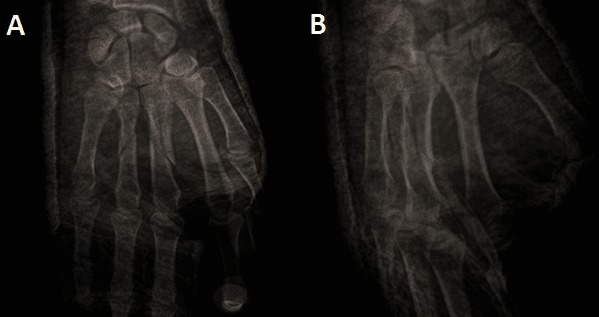
Plain view radiograph, **Fig. 1B** Oblique view radiograph. Both showing spiral and oblique fracture

A 35-year-old right-handed man sustained an injury to his right hand following a fall during roller-skating. He was reviewed in our clinic four days after the injury. The hand was splinted four days before in an orthopedic clinic after the plain radiography showed apparently non-dislocated stable shaft fractures of the third (spiral) and fourth metacarpal (oblique) (**Fig. 1A, 1B**).

**Fig. 2:**
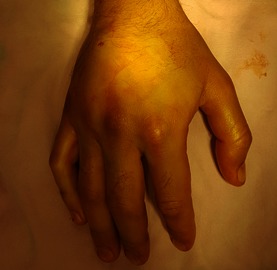
Clinical assessment of the hand at 4 days from injury

After the removal of the cast, the hand was swollen and extremely painful during active/ passive mobilization, worse than during the first day of injury (**Fig. 2**) in spite of the elevated position and local cold application. 

**Fig. 3:**
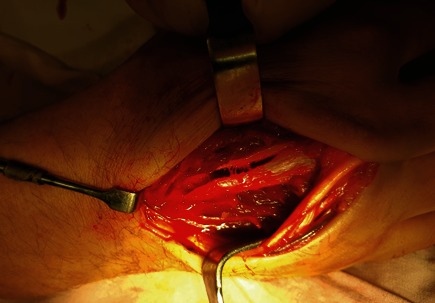
Spiral complex C1 fracture of the third metacarpal shaft

Surgical management was adopted. Under general anesthesia, a passive local exam of the hand was performed. The middle and the ring finger presented a rotational misplacement, 10° radial deviation for the third finger with a shortening and 10° ulnar deviation for the fourth finger. A dorsal “S” incision between the skin projection of the third and fourth metacarpal was performed, with a dissection down to the metacarpals. The periosteum was incised in order to expose the fractured bones. During the surgical exploration, the fractures were classified as spiral complex C1 for the third metacarpal shaft and fragmented wedge B3 of the fourth metacarpal according to AO classification (**Fig. 3**). 

**Fig. 4:**
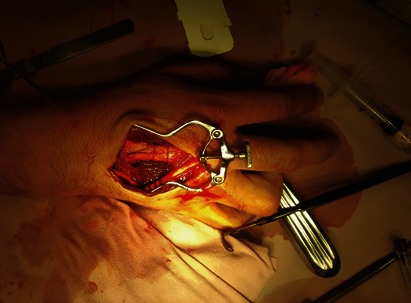
Temporary reduction using cerclage

**Fig. 5:**
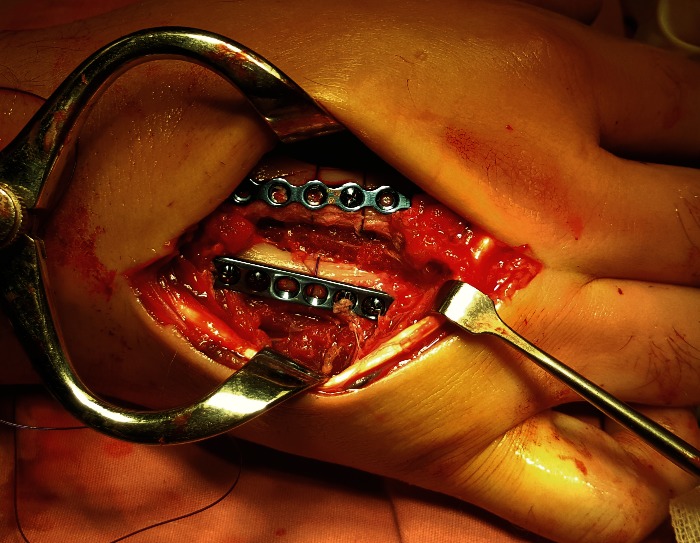
Definitive fixation using plates and screws

The fractures were reduced by using a 2-0 polypropylene cerclage (**Fig. 4**). A definitive fixation was performed by using a 0.8 mm profile height x 1.7 mm nine-hole plate with five self-tapping screws (1.7 mm x 10 mm) for the third metacarpal and by using a 0.8 mm height profile x 1.7 mm six hole-plate with four self-tapping screws for the other fracture (**Fig. 5**). 

**Fig. 6:**
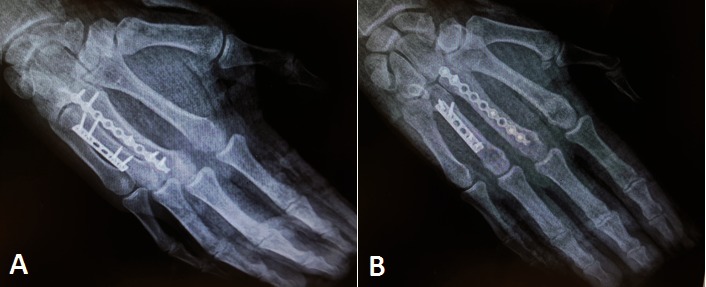
Oblique (A) and Plain (B) intraoperative radiographs

**Fig. 7:**
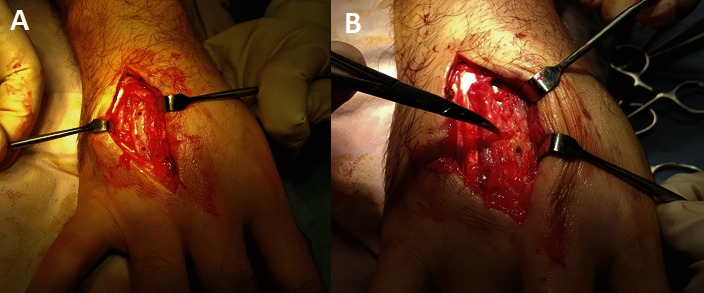
Removing the osteosynthesis implants from the forth (A) and third (B) metacarpal

**Fig. 8:**
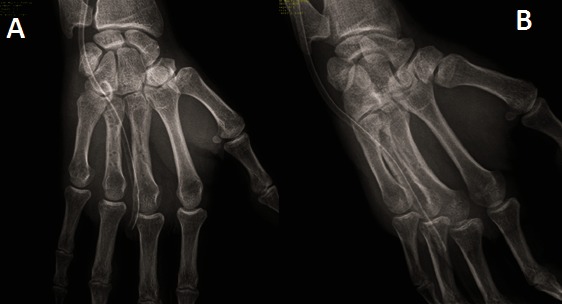
Postoperative plain (A) and oblique (B) radiographs

The cerclage was removed after fixation. The periosteum was removed on a small portion in order to install the plates; the rest of it being used to cover the osteosynthesis material to prevent tendon adhesion. After hemostasis, the skin was closed by using upholstery interrupted and intradermal continuous suture. There were no complications during surgery (**Fig. 6**). The fixation was protected by using a volar splint for the wrist, hand, and fingers. The hand and wrist were immobilized for 12 days to allow the skin and soft tissue to heal. The splint was removed and the patient started recovery sessions and came to periodic follow-up at every two weeks. After four months, the patient underwent surgery and the plates and screws were removed (**Fig. 7**,**8**). After removing the postoperative splint at 10 days, the patient followed twenty recovery sessions. The patient healed very well and fully recovered.

## Discussions

**Fig. 9:**
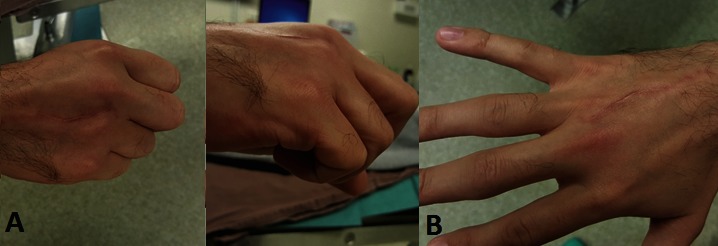
Clinical assessment after 4 months from surgery. A – complete flexion, B – complete extension

Our expectation was that this fixation method would be reliable in maintaining the fractures reduced and allowing an early range of motion, despite having to remove a part of the periosteum that could lead to complex regional pain syndrome. The outcomes were clinically assessed by using the Disability of Arm, Shoulder and Hand score (DASH score) and radiographically. The DASH score was 16.7 at 3 months. The ring finger was fully recovered, but the middle finger had a 30% function loss because of a 30° flexion deficit (Total Active Motion -TAM score was 205°). We expected this to occur because of the fracture site that was prolonged distally close to the capsular insertion and because of the long plate that was used in order to obtain a good reduction, leading to the shortening of the stretching range of the extensor tendon (**Fig. 9**). 

Another cause that could be problematic for the extensor mechanism is the shortening of the metacarpal, because of the insertion through the sagittal bands on the metacarpal head that leads to extensor lag. For every 2 mm of long axis shortening, there will be a 7° loss of extension [**4**]. Usually, there is a 20° extension loss tolerance, meaning that the metacarpal can be shorter, up to 5-6 mm, without creating a great extension deficit. Another important aspect that should be taken into consideration is the insertion of extensor carpi radialis longus and brevis. They are attached to the second and third finger metacarpal bases. The fourth finger has no proximal extensor attachment. In some cases, these tendons can lead to misplacement (dorsal angulation of the shaft) at the fracture site, due to their deforming forces. Dorsal angulation of the shaft is tolerated without a function loss if it is less than 15° for the ring and small finger, and less than 10° for the index and middle finger, due to the mobility of the carpal-metacarpal joints [**5**]. If the fracture site is distally located, close to the metacarpal neck, the fractures can be treated without a surgical reduction if the apex dorsal angulation is less than 45° at the small finger, 30° at the ring finger, 20° at the middle finger and 15° at the index finger [**6**,**7**]. Most studies showed that more than 30° angulation could lead to a weak grip of the hand [**8**]. There is a mechanism to compensate the flexion malposition in the metacarpals by gaining more extension range in the metacarpal phalangeal joint (MCP), but this could lead to an improper force discharge at the proximal interphalangeal joint (PIP), with extensor lag called pseudo clawing. It is important to correctly assess the extension of PIP when the MPC is fully flexed or extended. Another misplacement of the bone fragments that usually need surgical reduction and osteosynthesis implants is the rotational deformity. If the fingers are in extension, the rotational defect can be missed. It becomes more visible when the fingers are in flexion, therefore each degree of rotation of the shaft leads to 5° rotation at the tip of the finger and to 1.5 cm overlapping during closed fist [**9**]. This could lead to serious disability of the hands. It is important in order to correctly assess and to compare the injured part with the healthy one, where all the fingers should be orientated to the scaphoid tubercle. Additionally, the associated comorbidities can contribute to long-term prognosis and the onset of early complications, especially in patients with renal impairment (dialysed or not) that includes a complex and difficult postsurgical management (acute on kidney injury, acute-on-chronic kidney disease – hyperkalemia and arrhythmia, metabolic acidosis, anuria, etc.). Furthermore, an active medical management with a close assessment of all the parameters prior and after the intervention contributes to the outcome improvement [**10**-**21**].

In this case, there was only a shortening and a rotational deviation without a dorsal angulation of the metacarpal shaft. This could only be examined under general anesthesia because the pain did not allow a full flexion in order to assess the orientation of the tip of the fingers.

When the clinical assessment is not sufficient, angulation or rotational misplacements are in most cases confirmed during the radiological assessment. Usually, posterior-anterior, lateral, and oblique views are enough to establish the type of fracture. In order to obtain a better image of the second and third metacarpals, the half pronation oblique view can be used. The half supination view is more suited for the fourth and fifth metacarpals. In order to assess the metacarpal heads, the hand with the posterior aspect of the fingers is put against the X-ray panel with the elbow in extension and the MCP joints at 65° flexion. This is called the Brewerton views and it involves the positioning of the X-ray beam angled at 15° medial-to-lateral [**22**]. When available, computed tomography is the most suited to assess complex comminuted fractures.

Even if in most of the cases the radiological assessment is enough to confirm and establish the type of fracture, in this case, the fractures were misclassified. The fractures initially appeared as being reduced, with spiral and oblique fracture site. During surgery we explored and confirmed comminuted and displaced metacarpal fractures with four fragments, two proximally and two distally, with long axis shortening for the third and rotational misplacement for both.

Based on the radiological aspects, a non-operative treatment was an option for properly healing before surgery. After the clinical assessment, under general anesthesia, was performed, the rotational misplacement established the need of osteosynthesis in order to reduce and fixate the fractures. Intramedullary nail (Kirschner wire) or plates and screws were the best option. We decided to use plates and screws in order to allow an early motion, even if it is associated with a high risk of avascular necrosis, because it involves removing a small area of the periosteum [**23**]. After osteosynthesis with plates and screws, the risk of a complication to occur is between 32% and 36% [**12**]. According to the literature, stiffness is the most common with TAM less than 220°, followed by minor extensor lag, contractures, and major extensor lag [**24**]. Other complications described as being very rare, involve nonunion, osteomyelitis or tendon rupture.

Even so, during surgery, we agreed that this was the most suited option to reduce the comminuted fractures. The other option regarding the use of wire cerclage was less rigid. Intramedullary nailing was no longer an option to reduce this type of fracture.

At 4 months, we decided to remove the hardware in order to maximize the function recovery. The DASH score was 2.5 at six months and 1.7 at twelve months after twenty recovery sessions, regaining 95% of the middle fingers function.

## Conclusions

In order to reduce comminuted rigidly misplaced metacarpal fractures and to allow the early motion recovery osteosynthesis with plates and screws led to very good results, even if a small portion of the periosteum needed to be removed, therefore compromising the periosteal vascular network of the comminuted fragments. However, there are a series of complications that should be taken into consideration before choosing this method. In most of the cases, the radiological assessment offers enough information in order to classify a fracture, but the most certain diagnosis is established during exploratory surgery.

**Conflict of interests**

The authors declare no conflict of interests.
